# Microchromosome polymorphism in the sand lizard, *Lacerta agilis* Linnaeus, 1758 (Reptilia, Squamata)

**DOI:** 10.3897/CompCytogen.v10i3.7655

**Published:** 2016-09-08

**Authors:** Artem P. Lisachov, Pavel M. Borodin

**Affiliations:** 1 Institute of Cytology and Genetics, Russian Academy of Sciences, Siberian Department, Novosibirsk 630090, Russia; 2 Department of Cytology and Genetics, Novosibirsk State University, Novosibirsk, Russia

**Keywords:** Synaptonemal complex, Lacertidae, chromosome evolution

## Abstract

Most true lizards (Lacertidae) share a conservative karyotype, consisting of 18 pairs of macrochromosomes and one microchromosome pair. Homeologues of the microchromosome are present in other squamates and even in chickens. No structural autosomal microchromosome polymorphisms have been described previously in lizards. We found homozygous and heterozygous carriers of a microchromosome variant in a Siberian population of the sand lizard, *Lacerta agilis* Linnaeus, 1758. The variant microchromosome was almost twice as long as the standard one. In heterozygotes at pachytene, the microchromosomes firstly pair in proximal regions and the central part of the longer axial element undergoes foldback synapsis, then its distal region pairs with the distal region of the standard partner. At metaphase-I, the heteromorphic microchromosome bivalents have a proximal chiasma. The content of the additional segment was Ag-NOR, C-like DAPI, CMA3 negative. FISH with telomere PNA probe did not detect interstitial (TTAGGG)_n_ sequences in the heteromorphic and any other bivalents. Both homo- and heterozygous carriers were phenotypically normal. The presence of homozygotes shows that heterozygotes are fertile. Reduction in the number of microchromosomes is a clear trend in squamate evolution, as a result of microchromosomes fusing together or with macrochromosomes. Our findings indicate that gaining additional DNA may lead to a transformation of microchromosomes into small macrochromosomes without fusion.

## Introduction

Microchromosomes are considered as the part of the ancestral tetrapod genome (Uno et al. 2012). They have been conserved in different degrees in modern lineages. In amphibians, microchromosomes are present in primitive families of all three orders, independently disappearing in the more derived ones (Morescalchi 1980). Among amniotes, birds retain the most archaic karyotype with many microchromosomes, whereas in squamate reptiles the number of microchromosomes has decreased (Olmo 2008). They comprise a half or more of the chromosome sets in iguanids, agamids, snakes and varanids. However they are not present in crocodiles (Crocodilia) and geckos (Gekkota). It is believed that the independent and parallel loss of microchromosomes in these groups has been achieved by their fusion with each other and with macrochromosomes (Srikulnath et al. 2014, 2015). The true lizards (Lacertidae) also lost most of microchromosomes. Lacertidae are one of the most widespread reptile families. At the moment, 322 species are recognized within the family (Uetz, Hošek, The Reptile Database). Karyotypes of the true lizards are rather conservative. Most of them have 18 pairs of macrochromosomes and one pair of microchromosomes (2n=38) (Gorman 1969, Olmo and Signorino 2005). The microchromosome is usually much shorter than the smallest macrochromosome and can be unequivocally identified by size. Some species, such as *Zootoca vivipara* (Lichtenstein, 1823) and *Iberolacerta monticola* (Boulenger, 1905), do not have microchromosomes at all. Homeologues of this microchromosome have been found in snakes, varanids, agamids and even in chicken (Srikulnath et al. 2014).

Chromosome polymorphism in the lacertids is poorly known. We are aware of only one case of chromosome polytypism: variation in C-band distribution between subspecies and populations of Italian wall lizard *Podarcis siculus* (Rafinesque-Schmaltz, 1810) (Olmo et al. 1986). No examples of microchromosome morphology polymorphism have been described in true lizards.

In this paper we describe a long microchromosome variant which covers the gap between micro- and macrochromosomes. We found this variant in Siberian population of the sand lizard, *Lacerta agilis* Linnaeus, 1758, and examined its meiotic behavior in homo- and heterozygotes by fluorescent microscopy of synaptonemal complexes (SCs) and metaphase-I spreads using immunolocalization of SYCP3 (the major protein of the SC axial elements) and centromeres, and electron microscopy using Ag-NOR staining.

The SC analysis via electron microscopy and immunofluorescent staining is widely used in vertebrate cytogenetics (Lisachov et al. 2015; Wallace and Wallace 1995; Reed et al. 1990; Calderon and Pigozzi 2006; Basheva et al. 2014). At pachytene, the compactization of chromatin is much lower than at metaphase, and thus the SC analysis provides higher resolution than the conventional metaphase techniques. This is particularly useful in the microchromosome studies. The requirement of the homologous pairing at pachytene makes the SC analysis a perfect tool in studying any chromosomal heteromorphisms, both autosomal and gonosomal. Our work is the first in which the immunofluorescent staining is applied for reptiles.

## Materials and methods

The lizards were caught near Berdsk (54°46.37'N, 83°5.77'E) (ten specimens) and Novosibirsk (54°50.78'N, 82°57.92'E) (four specimens), Novosibirsk region, Russia. Trapping, handling, and euthanasia of animals were performed according to the protocols approved by the Animal Care and Use Committee at the Institute of Cytology and Genetics of the Russian Academy of Sciences. All institutional and national guidelines for the care and use of laboratory animals were followed. No additional permits are required for research on this non-listed species in Russia. The specimens were deposited in the research collections of the Institute of Cytology and Genetics of the Russian Academy of Sciences.

The spreads of spermatocytes were prepared according to the protocol of Peters et al. (1997).

For electron microscopic examination the spreads were stained with silver nitrate (Howell and Black 1980) and covered with plastic film. The spreads, after light microscopic examination, were transferred to specimen grids and examined with electron microscope JEM-100 (JEOL, Japan) at 80 kV.

Immunostaining was performed according to the protocol described by Anderson et al. (1999) with modifications. SCs were detected by rabbit polyclonal antibodies to the SC axial element protein SYCP3 (1:500, Abcam, Cambridge) and goat anti-rabbit Cy3-conjugated secondary antibodies (1:500, Jackson, West Grove). Centromeres were detected by human anti-centromere antibodies (ACA) (1:100, Sigma-Aldrich) and goat anti-human FITC conjugated secondary antibodies (1:100, Vector Laboratories). All antibodies were diluted in PBT (3% bovine serum albumin and 0.05% Tween 20 in 1xPBS). A solution of 10% PBT was used for blocking unspecific antibody binding. Primary antibody incubation was performed overnight in a humid chamber at 37 °C, and secondary antibody incubation was performed for 1 h at 37 °C. Finally, slides were mounted in Vectashield with DAPI (Vector Laboratories) to stain DNA and reduce ﬂuorescence fading. The spreads were photographed using an Axioplan 2 Imaging (Carl Zeiss) microscope with CCD camera (CV M300, JAI Corporation, Japan), CHROMA filter sets, and ISIS4 image processing package (MetaSystems GmbH, Germany). The length of each bivalent in the spread was measured using MicroMeasure 3.3 software. Statistica 6.0 software package (StatSoft, Tulsa, OK, USA) was used for descriptive statistics.

The heterochromatic regions were visualized by a previously described C-like DAPI staining technique (Lisachov 2013). The coverslips were carefully removed after the photographs were taken. The preparations were washed in 2×SSC for 5 min to remove the antifade solution and then dehydrated in ethanol series 70%, 80% and 100% for 3 minutes in each. The preparations were then air-dried and kept in 0.2 N HCl at room temperature for 20 min to 30 min. The slides were transferred to saturated barium hydroxide solution at 55 °C for 1 min to 10 min. The preparations were then incubated in 2×SSC at 55 °C to 60 °C for 60 min. The preparations were re-mounted in the antifade solution with DAPI.

For chromomycin A_3_ (CMA_3_) staining, we used the solution of 0.4 mg/ml CMA_3_ and 0.01 M MgCl_2_ in PBS. After preparing, the solution was left to stabilize at +4 °C for two days. Then 25 µl of the solution was put onto the slide already subjected to immunostaining, and covered by the coverslip. After 1 h, the slide was washed in PBS for 5 min and then mounted in the antifade solution with DAPI. After staining, the slide was again left to stabilize at the room temperature in the dark for three days.

The telomeric (TTAGGG)_n_ sequences were detected with a commercial FITC-conjugated PNA probe (LifeTechnologies) according to the manufacturer’s protocol.

## Results

**Figure 1.**
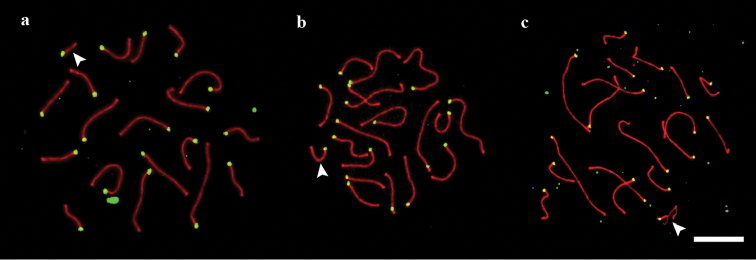
SC spreads of sand lizards. **a** standard karyotype **b** homozygote for the long variant of SC 19 **c** heterozygote for the long variant of SC 19. Arrowheads indicate SC 19. Red: SYCP3. Green: ACA. Scale bar: 10 µm.

**Figure 2.**
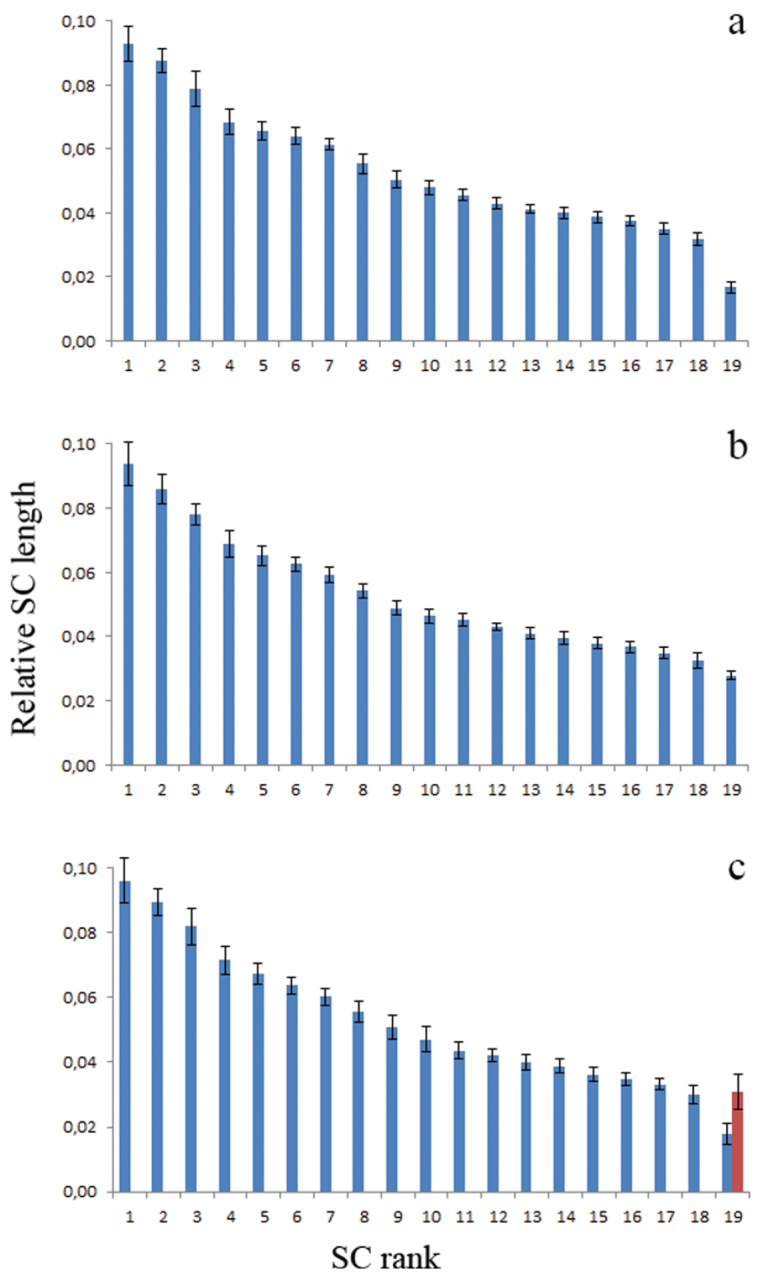
Relative lengths of the SCs in three sand lizards. **a** standard karyotype (21 spreads) **b** homozygote for the long variant of SC 19 (22 spreads) **c** heterozygote for the long variant of SC 19 (18 spreads). Red column: the long variant in the heterozygote. Bars show standard deviation.

**Figure 3.**
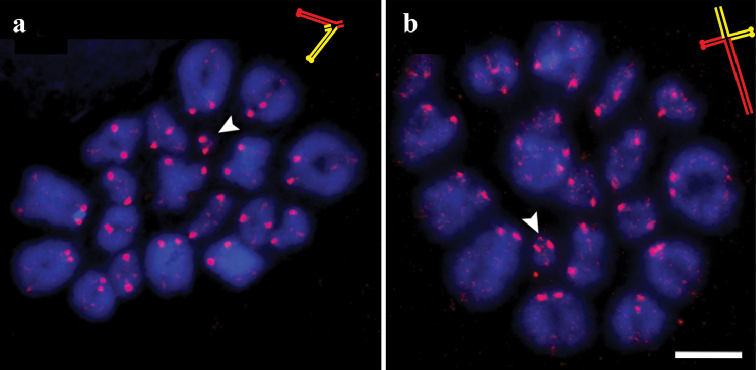
Meiotic metaphase I in sand lizards. **a** standard karyotype **b** heterozygote for the long variant of SC 19. Arrowheads and schematic inserts show bivalent 19. In schematic inserts red and yellow colors show the homologues. Red: SYCP3. Blue: DAPI. Scale bar: 10 µm.

We examined 14 male lizards, ten from Berdsk and four from Novosibirsk. In all of them, 19 acrocentric bivalents were seen at synaptonemal complex spreads and at metaphase I plates (2n=38). The mean total length of the SCs was 178±21 µm. The macroSCs formed a gradually decreasing set. In five individuals from Berdsk and the four from Novosibirsk, the microchromosome (SC 19) was significantly smaller than the smallest macrochromosome (SC 18). Their mean sizes, relative to the total SC length, were 1.68±0.14% and 3.14±0.31% respectively (P <0.001)). The microchromosome was thus easily identifiable at SC spreads, as well as at meiotic metaphase I plates (Figs 1a, 2a, 3a, 5a–c).

In one Berdsk individual (#3) the difference between SC 18 and SC 19 was less pronounced although still significant, 3.24±0.24% and 2.78±0.14% respectively (P <0.001) (Figs 1b, 2b, 5d–f).

**Figure 4.**
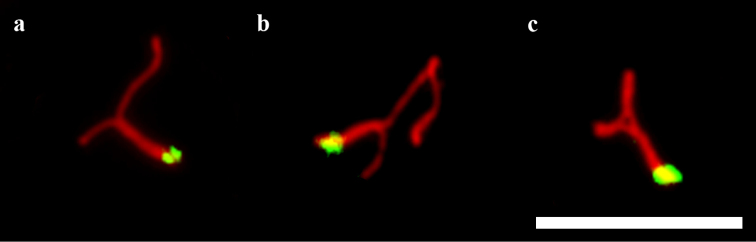
Synaptic configurations of the heteromorphic SC 19. **a** synapsis in the proximal regions **b** the longer element showing foldback self-synapsis **c** completely paired forming a T-shaped configuration. Scale bar: 5 µm.

In four Berdsk individuals ##1, 7, 9, 10 we detected a heteromorphic SC 19 with one axial element element significantly longer than the other (P=0.03). The relative length of the longer element was 3.08±0.53% of the total macrochromosomal SC length plus the long element 19. The shorter element was 1.77±0.33% of the total macrochromosomal SC length plus the short element 19 (Figs 1c, 2c, 4).

Fig. 4 shows variability of the synaptic configurations of the heteromorphic SC 19, which probably reflects the sequence of conjugation. At early stage of conjugation the proximal parts of the elements were synapsed while the distal ones remained unpaired (Fig. 4a). At later stages, the longer element formed a foldback in the middle (Fig. 4b). Finally, the distal ends of the elements became synapsed, forming a T-like structure (Fig. 4c).

An interesting feature of the metaphase I bivalents of the sand lizard is that they retain some traces of SYCP3 (Fig. 3), which is more pronounced at the centromeres. A strong SYCP3 signal is usually co-localized with the centromere signal (Suppl. material 1). We found five good metaphase I plates in a specimen with heteromorphic bivalent 19. In all of them the smallest bivalent had one proximal chiasma and asymmetric distal ends (Fig. 3b). This may indicate that recombination in the heteromorphic bivalent usually occurs in the proximal region.

**Figure 5.**
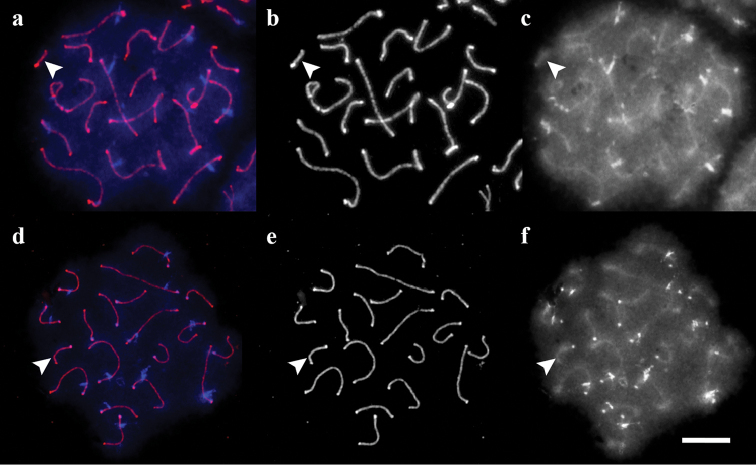
SC spreads of the sand lizards after C-like DAPI staining. **a–c** standard homozygote **d–f** homozygote for the long variant of chromosome 19 **a, d** merged images **b,e** SYCP3 **c, f** DAPI. Arrowheads show SC 19. Scale bar: 10 µm.

Insertions or/and amplifications of C-positive chromatin have been suggested as common causes of an increase in chromosome size (Agulnik et al. 1993). To test this, we carried out C-like DAPI staining (Lisachov 2013) in the carriers of the enlarged variant of the chromosome 19. The centromeres of all chromosomes were C-positive. We also detected pericentromeric C-bands in 12 large chromosomes. A similar pattern has been observed in the Iberian rock lizard *Iberolacerta monticola* (Giovannotti et al. 2014). However, we did not find an interstitial C-band in either long or standard variants of the chromosome 19 (Fig. 5).

**Figure 6.**
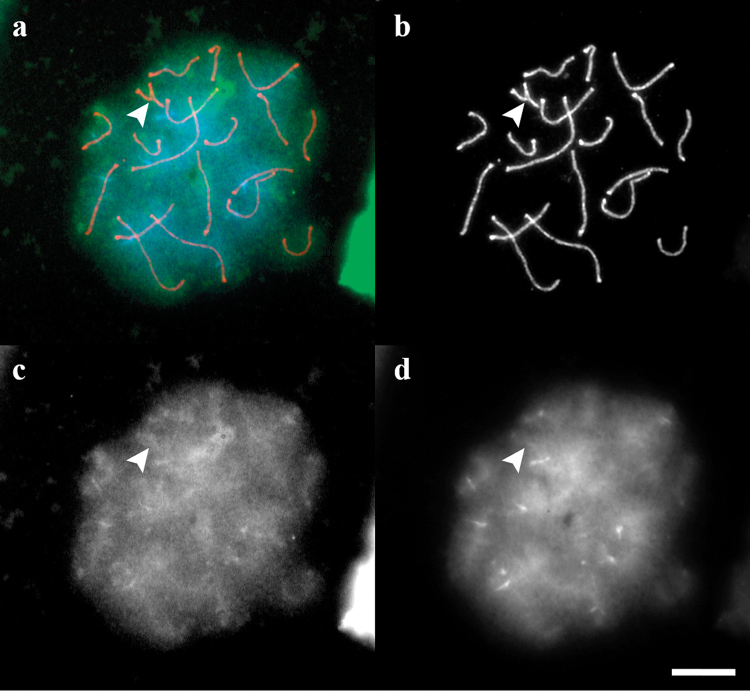
CMA3 and DAPI staining of the SC spread from a heterozygous individual. **a** merged image **b** SYCP3 **c** CMA3 **d** DAPI. Arrowhead shows SC 19. Scale bar: 10 µm.

To test whether there is an accumulation of GC-rich sequences in the long variant of the chromosome 19, we used CMA_3_ staining. This fluorochrome mostly gave a uniform fluorescence along all the chromosomes, including the enlarged chromosome 19 (Fig. 6). When present, the differential staining repeated the DAPI staining pattern.

**Figure 7.**
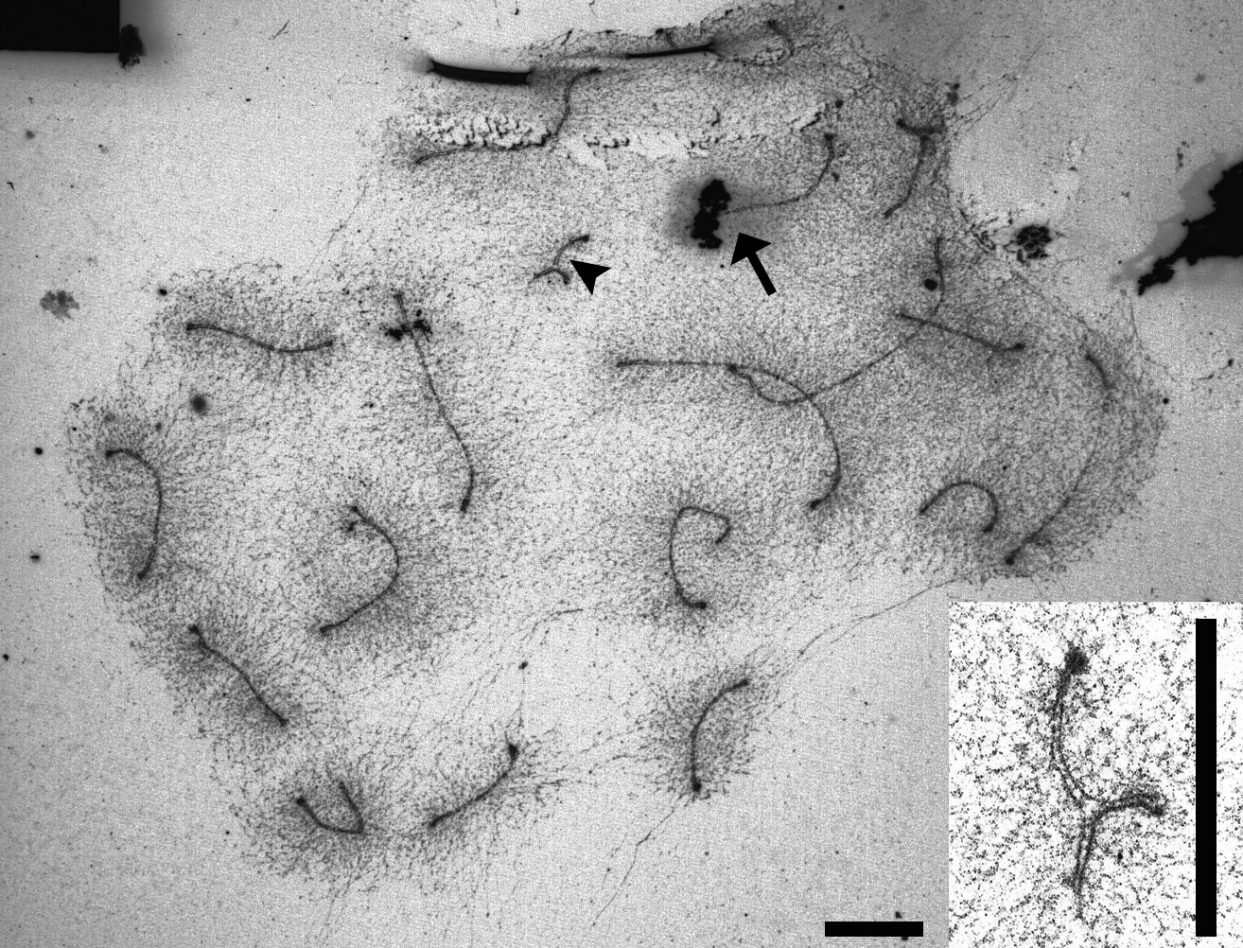
Ag-NOR stained SC spread of the sand lizard heterozygous for the long variant of chromosome 19. The arrowhead and insert show heteromorphic SC 19. The arrow shows NOR at a macrochromosome. Scales bars: 5 µm.

The telomeric sequences are known to extensively accumulate at the W chromosome of *Lacerta agilis* (Matsubara et al. 2015). To test whether this sequence is responsible for the enlargement of the chromosome 19, we carried out FISH with the FITC-labeled PNA probe after immunostaining. The telomeric signals occurred only at the terminal parts of the chromosomes, and no extensive accumulation was present at the enlarged variant of the chromosome 19 (Suppl. material 2). The nucleolus organizer (NOR) amplification or transposition might be the cause of chromosome elongation (Woznicki et al. 1998). Ag-NOR staining revealed a single NOR distally at one of the medium chromosomes (1st to 6th in the set), but nothing at chromosome 19 (Fig. 7) This NOR location is consistent with the previous findings (Vujošević and Blagojević 1999).

## Discussion

We have concluded that the carriers of the heteromorphic SC 19 (specimens ##1, 7, 9, 10) were heterozygotes for the long variant of the chromosome 19, while the specimen #3 was homozygous for this variant, and all other individuals were homozygous for the standard variant described for this species previously. Since similar karyotypes consisting of 18 macrochromosome pairs and 1 microchromosome pair are characteristic for most other lacertid species (Olmo and Signorino 2005), the short microchromosome is presumably ancestral, and the long variant described here originated from it by a chromosomal rearrangement.

Based on the synaptic configurations observed in the heterozygote, we suggest that the long variant of the microchromosome probably contains a palindromic sequence in its median region. This sequence shows foldback self-synapsis (Figs 1c, 4b-c, 7). Foldback synapsis is not necessarily connected with palindromes. For example, in the iguanian lizard *Sceloporus graciosus* Baird & Girard, 1852, which has sex chromosomes differentiated in length, the longer element also forms a lateral buckle. But in *Lacerta agilis* the longer element starts to form a self-paired buckle when normal pairing is present only at one end, and is not yet constrained by the difference in length. In contrast, in the *Sceloporus graciosus* the buckle forms as the result of synaptic adjustment, when the axial elements are anchored at the ends and try to pair completely, compensating the difference in length (Reed et al. 1990). We examined a possibility that the additional region of the long variant is composed of repeated sequences. Our tests for NOR, (TTAGGG)_n_, AT- and GC-rich repeats gave negative results. All major satellites which are characterized in the Lacertidae so far are AT-rich (Ciobanu et al. 2004). In *Iberolacerta monticola*, the satellites are located in the centromeric and pericentromeric DAPI/C-positive bands (Giovannotti et al. 2014). These bands are similar to the bands seen in our samples (Fig. 5). Moreover, the *TaqI* satellite of *Iberolacerta monticola* was found to have homologues in *Lacerta* species. Therefore, we suggest that the elongation of the chromosome 19 is not due to the accumulation of the known *Lacerta agilis* satellites.

The polymorphic variant of the microchromosome seems not to affect the fitness of the carriers. Homozygous and heterozygous carriers of the variant were phenotypically normal compared with the specimens having the normal karyotype. The occurrence of the homozygote and the presence of the mature spermatids on the preparations from the heterozygotes (not shown) indicate that heterozygotes are fertile.

The five carriers of the long microchromosome were found in the same area of several thousand square meters of grassy river terrace slope, between a motorway and a river. This variant is possibly local and shared by a group of related animals. If this is the case, a homozygote can be produced in the third generation after the origin of the variant chromosome.

Fusion of microchromosomes with each other and with macrochromosomes is considered as the main mechanism of the reduction of the number of the microchromosomes (Uno et al. 2012). However, our finding indicates that enlargement of a microchromosome by gaining additional DNA content can close the gap between micro- and macrochromosomes, and this can be an alternative route for microchromosome transformation. This may not be rare, since, in several reptile lineages, loss of the microchromosomes does not lead to a decrease in chromosome number (Olmo and Signorino 2005). Perhaps, this Siberian population of the sand lizard gives an insight of how the transition to all-macrochromosome karyotype might occur.

## Conclusion

We found a polymorphic variant of the 19^th^ chromosome in one population of the sand lizard, *Lacerta agilis*. It is presented in both heterozygous and homozygous states, and the carriers seem to be phenotypically normal and fertile. The polymorphic variant is two-fold larger than the normal one. Its exact content is unknown. We suggest that enlargement of an individual microchromosome by accumulating repetitive and other sequences may serve as alternative way in the process of the disappearance of the microchromosomes, along with the fusion events.

## Supplementary materials

Supplementary material 1 The colocalization of dense SYCP3 signal with the centromeres at meiotic metaphase I in the sand lizard Data type: Tif file Explanation note: The colocalization of dense SYCP3 signal with the centromeres at meiotic metaphase I in the sand lizard. Blue: DAPI, red: SYCP3, green: centromere. Scale bar: 5 µm. This dataset is made available under the Open Database License (http://opendatacommons.org/licenses/odbl/1.0/). The Open Database License (ODbL) is a license agreement intended to allow users to freely share, modify, and use this Dataset while maintaining this same freedom for others, provided that the original source and author(s) are credited.Artem P. Lisachov, Pavel M. Borodin

Supplementary material 2 FISH with the telomeric probe on the SC spread of the sand lizard heterozygous for the long variant of chromosome 19 Data type: Tif file Explanation note: FISH with the telomeric probe on the SC spread of the sand lizard heterozygous for the long variant of chromosome 19. Blue: DAPI, red: SYCP3, green: telomeric probe. Insert and arrowhead shows SC 19. Scale bar: 10 µm. This dataset is made available under the Open Database License (http://opendatacommons.org/licenses/odbl/1.0/). The Open Database License (ODbL) is a license agreement intended to allow users to freely share, modify, and use this Dataset while maintaining this same freedom for others, provided that the original source and author(s) are credited.Artem P. Lisachov, Pavel M. Borodin
