# Supplementary figures and images for: Microchromosome polymorphism in the sand lizard, Lacerta agilis Linnaeus, 1758 (Reptilia, Squamata)
Source: Comp Cytogenet. 2016 Sep 8;10(3):387–99. doi: 10.3897/CompCytogen.v10i3.7655 (PMC5088351; doi:10.3897/CompCytogen.v10i3.7655)

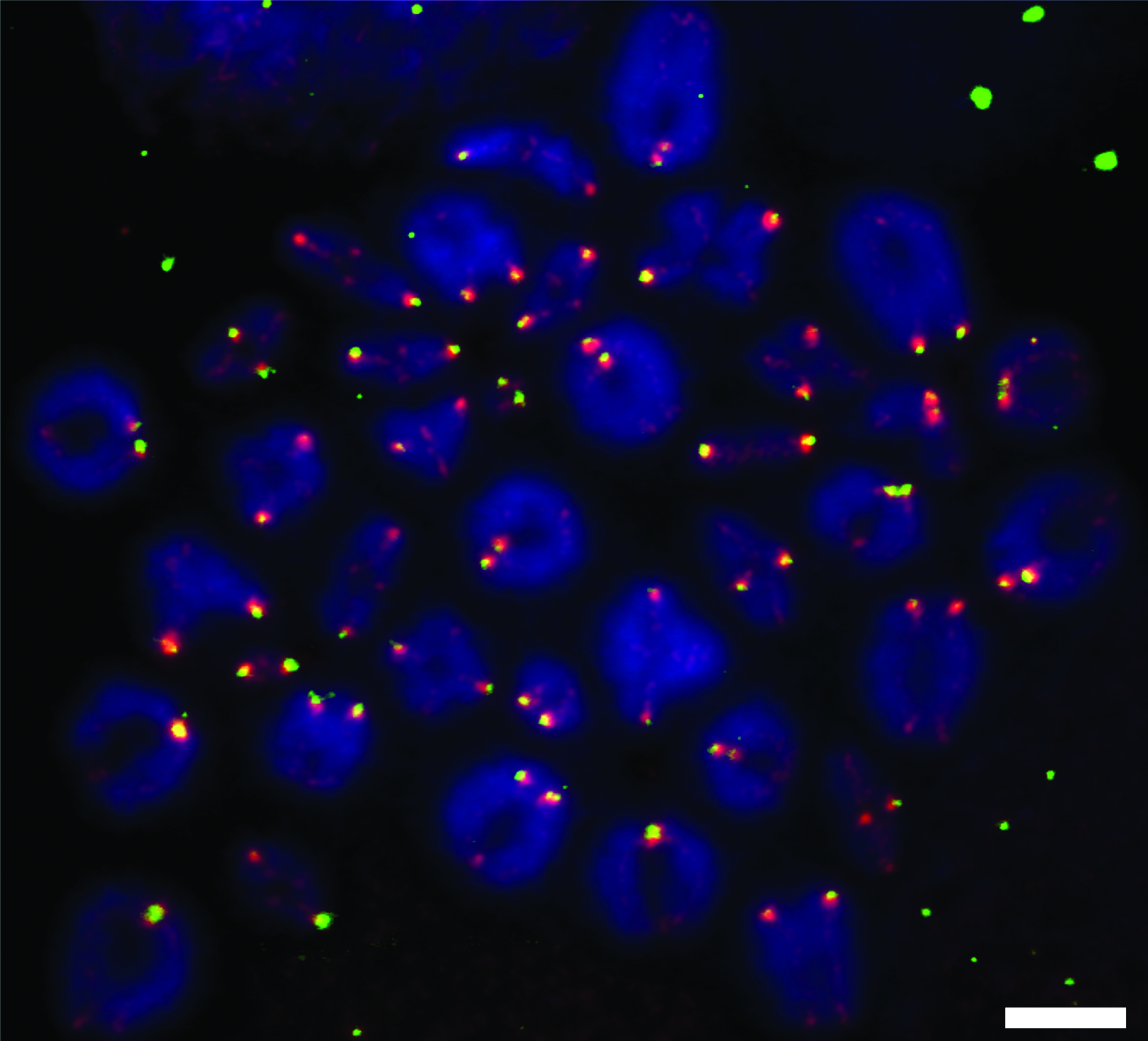

Supplement: Supplementary material 1 — The colocalization of dense SYCP3 signal with the centromeres at meiotic metaphase I in the sand lizard [file CompCytogen-010-387-s001.tif]

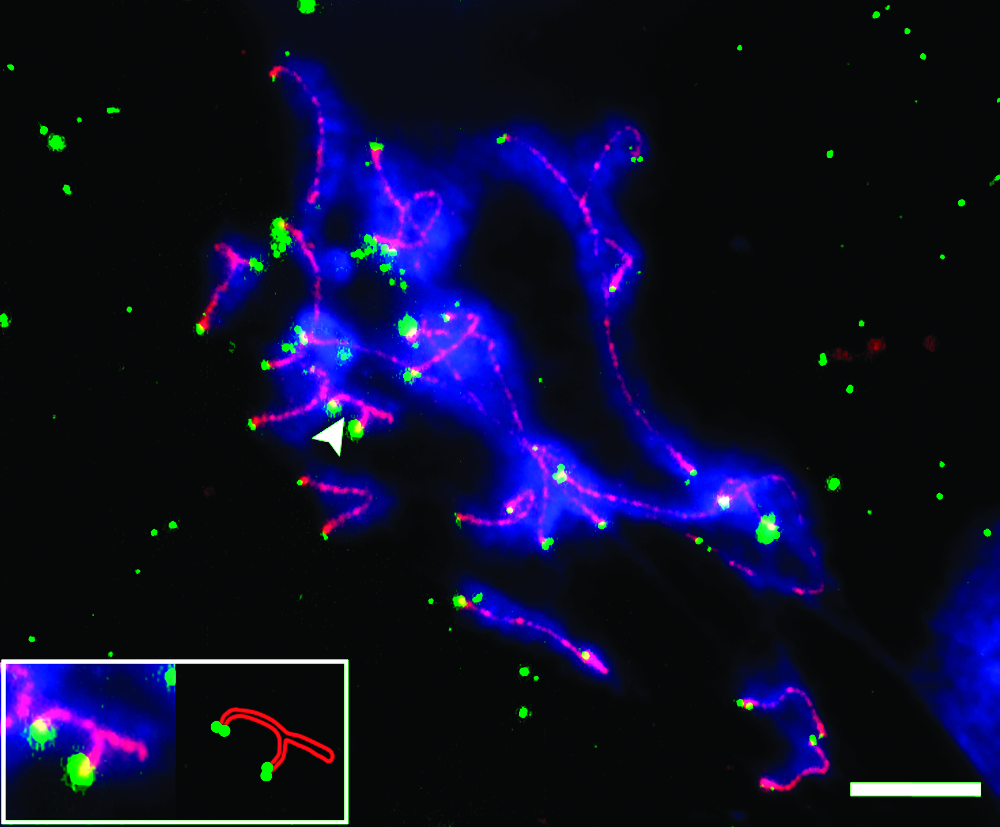

Supplement: Supplementary material 2 — FISH with the telomeric probe on the SC spread of the sand lizard heterozygous for the long variant of chromosome 19 [file CompCytogen-010-387-s002.tif]
